# A PTEN-Autophagy Risk Model for the Prediction of Prognosis and Immune Microenvironment in Hepatocellular Carcinoma

**DOI:** 10.1155/2023/2973480

**Published:** 2023-02-20

**Authors:** Fei Huang, Dilinaer Yaermaimaiti, Guanxin Ding, Lijun Zhao, Jing Zhou, Shunhua Wu

**Affiliations:** ^1^Department of Occupational and Environmental Health, School of Public Health, Xinjiang Medical University, Urumqi 830011, China; ^2^Department of Pathology, The Affiliated Tumor Hospital of Xinjiang Medical University, Urumqi 830011, China; ^3^Department of Epidemiology and Health Statistics, School of Public Health, Xinjiang Medical University, Urumqi 830011, China

## Abstract

**Background:**

The clinical behavior and molecular mechanisms of hepatocellular carcinoma (HCC) are complex and highly variable, limiting the discovery of new targets and therapies in clinical research. Phosphatase and tensin homolog deleted on chromosome 10 (PTEN) is one of the tumor suppressor genes. It is of great interest to discover the role of unexplored correlation among PTEN, the tumor immune microenvironment, and autophagy-related signaling pathways and to construct a reliable risk model for prognosis during HCC progression.

**Method:**

We first performed differential expression analysis on the HCC samples. By using Cox regression and LASSO analysis, we determined the DEGs contributing to the survival benefit. In addition, the gene set enrichment analysis (GSEA) was performed to identify potential molecular signaling pathways regulated by the PTEN gene signature, autophagy, and autophagy-related pathways. ESTIMATE was also employed for evaluating the composition of immune cell populations.

**Results:**

We found a significant correlation between PTEN expression and the tumor immune microenvironment. The low-PTEN expression group had higher immune infiltration and lower expression of immune checkpoints. In addition, PTEN expression was found to be positively correlated with autophagy-related pathways. Then, differentially expressed genes between tumor and tumor-adjacent samples were screened, and 2895 genes were significantly associated with both PTEN and autophagy. Based on PTEN-related genes, we identified 5 key prognostic genes, including BFSP1, PPAT, EIF5B, ASF1A, and GNA14. The 5-gene PTEN-autophagy risk score (RS) model was demonstrated to have favorable performance in the prediction of prognosis.

**Conclusion:**

In summary, our study showed the importance of the PTEN gene and its correlation with immunity and autophagy in HCC. The PTEN-autophagy.RS model we established could be used to predict the prognosis of HCC patients and showed significantly higher prognostic accuracy than the TIDE score in response to immunotherapy.

## 1. Introduction

Liver cancer is the second-most deadly cancer and also results in increased incidence and mortality annually worldwide [1]. Hepatocellular carcinoma (HCC) is the most common type of liver malignancies [2]. The clinical behavior and molecular mechanisms of HCC are complex and highly variable, limiting the discovery of new targets and therapies in clinical research. There is an urgent unmet need to explore the potential mechanisms associated with HCC progression to improve the clinical diagnosis, optimize the treatment strategy, and predict prognosis.

As reported, phosphatase and tensin homolog deleted on chromosome 10 (PTEN) is one of the tumor suppressor genes that can negatively regulate the phosphatidylinositol 3-kinase (PI3K) pathway [3, 4]. The activated PI3K can convert phosphatidylinositol bisphosphate (PIP2) into phosphatidylinositol trisphosphate (PIP3), thereby activating downstream AKT/mTOR signaling pathways [4–6]. The PI3K/AKT/mTOR signaling pathways play important roles in cell growth and survival [7–9], driving tumor proliferation and progression. PTEN induces tumor suppression by dephosphorylating PIP3 that prevents the activation of the PI3K-AKT-mTOR signaling pathways. Recent studies have demonstrated that PTEN is the negative regulator of oncogene signaling pathways and is involved in immune regulation in the tumor microenvironment [10, 11]. Immunotherapy is an emerging promising component of many cancer treatment regimens that revives immune activation by blocking immune checkpoints, increases T cell infiltration, and enhances the antigen-presenting capability in the tumor microenvironment [12, 13]. It is therefore of great interest to study the correlation between PTEN and the tumor immune microenvironment during HCC progression.

Autophagy is a protein degradation system in which a dynamic response of cells to stress can be typically observed [14, 15]. During the process, cellular proteins and organelles are delivered to lysosomes and digested by lysosomal hydrolases, allowing cells to maintain homeostasis [16]. In normal cells, autophagy is active at the basal level which has an important homeostatic function in the ubiquitin proteasome degradation signaling pathway and maintains protein and organelle quality control [17, 18]. Autophagy can be further increased for pathogens and engulfment of apoptotic cells [19]. The association between autophagy and PTEN has been revealed in different cancers. It has been shown that PTEN can increase autophagy in human glioma cells [20] and breast cancer cells [21]. PTEN was considered as molecular switch node regulating autophagy and cancer metabolic reprogramming in the tumor microenvironment [22]. However, the link between PTEN and autophagy in the HCC tumor microenvironment has not been fully understood.

Herein, in this study, we collected HCC patient samples from The Cancer Genome Atlas (TCGA) dataset, which has been used for identifying gene signature in children with stage III acute lymphoblastic leukemia [23] and analyzed the difference in PTEN gene expression in liver tumor tissue and adjacent tissue, copy number variation (CNV) and single-nucleotide variant (SNV) mutation, methylation, and some other factors. Then, we explored the association between the PTEN gene and the tumor immune microenvironment through analyzing different immune cell scores. In addition, the correlation between autophagy and autophagy-related pathways, the significance of autophagy in tumorigenesis, and PTEN gene expression and mutation were analyzed. Considering the importance of the PTEN gene and autophagy in HCC, we screened 5 genes (BFSP1, PPAT, EIF5B, ASF1A, and GNA14) associated with both the PTEN gene and autophagy by correlation analysis, univariate cox analysis, LASSO, and multivariate cox analysis. Finally, the PTEN-autophagy.RS model was constructed, and we demonstrated that the model can further improve the prognosis of patients. By comparing the data of immunotherapy with the performance of TIDE, we confirmed that the PTEN-autophagy.RS model was more sensitive than the TIDE score in response to immunotherapy.

## 2. Materials and Methods

### 2.1. The Acquisition of HCC Datasets

The mutation data, copy number variation data, and RNA-seq data for HCC patients were downloaded through the TCGA GDC API. Samples without survival time or survival status were removed. The methylation data for HCC patients was downloaded through TCGA GDC API. The expression profile data of GSE76427 were downloaded from NCBI's Gene Expression Omnibus (GEO) website (https://www.ncbi.nlm.nih.gov/geo/).

### 2.2. Preprocessing of Methylation Data and RNA-Seq Data

For the TCGA RNA-seq data, we first removed samples without clinical follow-up information including survival time and survival status. After that, we converted ensemble to a gene symbol and took the average of the expressions with multiple gene symbols. Then, the base 2 logarithm of the expression file (transcript per million, TPM) was performed. After screening, a total of 360 primary tumor samples and 50 tumor-adjacent samples were included in the TCGA dataset.

For GEO data, we first removed the normal tissue samples and converted the probes into gene symbols using the platform annotation file. Then, we removed the mean of multiple gene names corresponding to one probe and one gene name corresponding to multiple probes. Next, the samples without clinical follow-up information such as survival time and survival status were also removed for further analysis. 115 tumor samples and 31,425 genes were finally screened.

For the methylation data in the TCGA dataset, we used the KNN function in the R package impute to complete the NA values and converted the beta value to an *M* value. After that, the cross-reactive CpG sites in the genome were removed according to the cross-reactive sites provided by a previous study [24]. Then, we removed the unstable genomic methylation sites including the removal of CpGs sites and single nucleotide sites on sex chromosomes.

### 2.3. Calculation of Immune Cell Infiltration Abundance in TME

We obtained the characteristic genes of 28 immune cells according to a previous study [25] and calculated the scores of 28 immune cells using the ssGSEA algorithm [26]. Meanwhile, we also used the CIBERSORT [27] to calculate the immune cell scores of each sample. The ESTIMATE software [28] was utilized to calculate the proportion of immune cells.

### 2.4. Gene Set Enrichment Analysis (GSEA)

The autophagy-related pathways and immune-related pathways were downloaded from the Molecular Signature Database (https://www.gsea-msigdb.org/gsea/msigdb/index.jsp) [29]. We performed ssGSEA to calculate the enrichment score of autophagy and immune-related pathways.  *P* value < 0.05 was determined as statistically significant. The correlation between PTEN and autophagy-related pathways was examined by employing Pearson correlation analysis.

### 2.5. Establishment and Validation of the PTEN-Autophagy.RS Mmodel

We first selected differentially expressed genes with prognostic significance, and the number of genes was further reduced by least absolute shrinkage and selection operator (LASSO) regression to obtain phenotype-related prognostic genes. The Akaike Information Criterion (AIC) was utilized for the regression analysis, which considered the number of parameters applied for fitting by stepAIC and the statistical fit of the model. We then calculated the risk score for each patient sample using the following formula: RiskScore = Σ*βi* × Exp*i*, where Exp*i* refers to the gene expression level of the phenotypic prognosis-related gene signature and *β* is the Cox regression coefficient of the corresponding gene. The risk score was determined for each HCC sample and was converted to a z-score. According to the threshold *z*-score of  0, the patient samples were divided into high- and low-risk groups. The Kaplan–Meier method was used for prognostic analysis, and the log-rank test was used to determine the significance of the difference.

### 2.6. Prediction of Responsiveness to the Immunotherapy Effect

We used the TIDE algorithm (http://tide.dfci.harvard.edu/) [30] to evaluate the TIDE score of the immunotherapy effect and verify the prediction of clinical responsiveness to the PTEN-autophagy.RS model. The immunotherapy dataset IMvigor210 [31] and the GSE91061 (https://www.ncbi.nlm.nih.gov/geo/query/acc.cgi?acc=GSE91061) dataset were included to evaluate the risk model. All the data of IMvigor210 were accessed through the IMvigor210CoreBiologies R package (http://research-pub.gene.com/IMvigor210CoreBiologies).

### 2.7. Statistical Analysis

The data were expressed as the mean ± standard deviation (s.d.). Statistical significance was determined by the Wilcox test through R software when comparing different groups. Kaplan–Meier curves were plotted, and the log-rank Mantel–Cox test was used for survival curve analysis.

## 3. Results

### 3.1. The Role of the PTEN Gene in Hepatocellular Carcinoma

**Figure 1:**
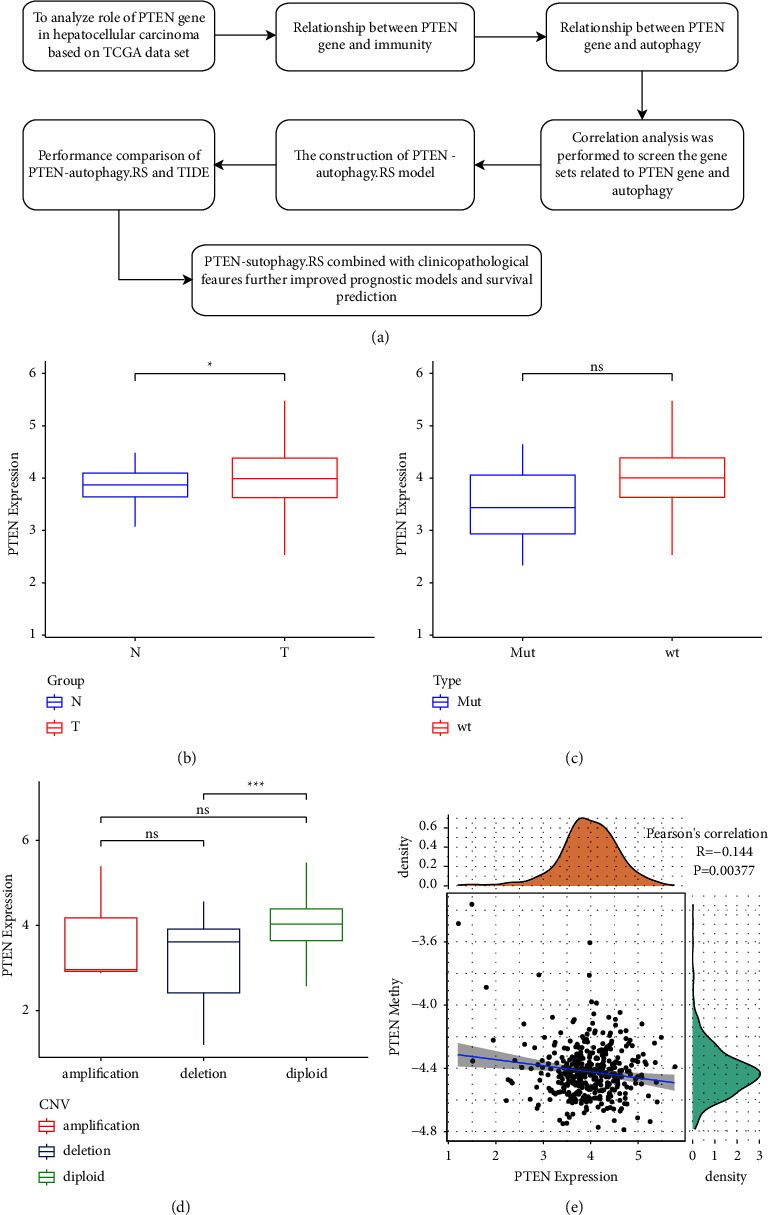
(a) The work flow of this study; (b) comparison of PTEN gene expression in tumor tissues (*n* = 360) and tumor-adjacent tissues (*n* = 50); (c) comparison of PTEN gene expression between samples with SNV mutation in PTEN (*n* = 8) and samples without SNV mutation (*n* = 352); (d) comparison of PTEN gene expression in different CNV groups including amplification (*n* = 3), diploid (*n* = 333), and deletion (*n* = 23); and (e) correlation analysis of PTEN gene expression and PTEN gene methylation.

The work flow of this study is shown in Figure 1(a). First, we analyzed the expression difference of the PTEN gene in the HCC tumor tissue samples and their adjacent tissues in the TCGA dataset. As shown in Figure 1(b), PTEN is highly expressed in the tumor tissue compared to adjacent tissue. We also found that a total of 8 samples were mutated in the PTEN gene among 360 samples. Although the gene expression of HCC samples without PTEN mutation was higher than that of samples with PTEN mutation, there was no significant difference between the two groups, which might be caused by the small sample numbers of the mutated PTEN gene group (Figure 1(c)).

Next, we divided tumor samples into three groups amplification, (deletion and diploid groups) according to the CNV status of PTEN including amplification, and we found that the samples without a CNV mutation in PTEN were significantly higher than the censored samples (Figure 1(d)). Furthermore, the correlation between the expression and the methylation of the PTEN gene was calculated, which showed a weak negative correlation (Figure 1(e)), suggesting that methylation might play a role in the regulation of gene expression.

### 3.2. The Relationship between the PTEN Gene and the Tumor Immune Microenvironment

**Figure 2:**
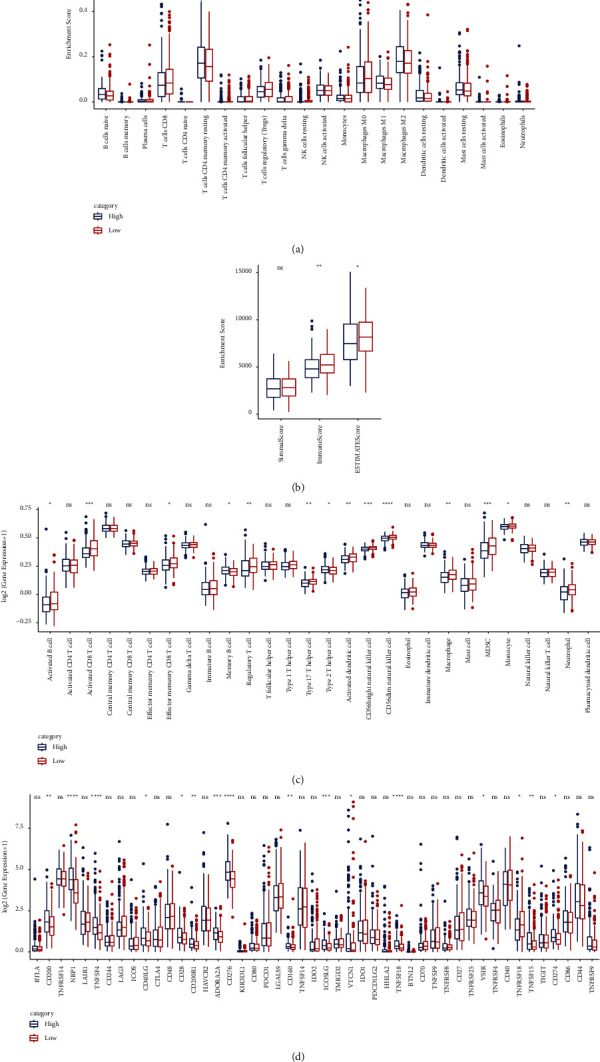
The immune characteristics of high (*n* = 180) and low (*n* = 180) PTEN expression groups in the TCGA dataset. (a) Comparison of 22 immune cell scores with high and low PTEN expression; (b) comparison of immune infiltration with high and low PTEN expression; (c) comparison of 28 immune cell scores with high and low PTEN expression; and (d) comparison of the levels of immune checkpoints with high and low PTEN expression.

To evaluate the relationship between the PTEN gene and immunity in HCC patients, we first divided the PTEN high-expression and low-expression groups by the median expression level and then calculated the scores of 22 immune cells in the different groups. It was found that the score of CD4 T cells and Treg cells in the low PTEN expression group was much higher than in the high-expression group (Figure 2(a)). The immune score of tumor samples was determined, and here the results showed a higher immune infiltration in the low-expression group compared to the high-expression group (Figure 2(b)). These results indicated that the PTEN gene might regulate the immune cell composition and infiltration into HCC tumor tissues.

According to the previous study [25], we collected the characteristic genes of 28 immune cells and calculated the scores of these immune cells. As shown in Figure 2(c), compared to the high-expression group, the PTEN low-expression group had much higher scores in some immune cells, especially CD8 T cells, Natural Killer (NK) cells, B cells, and macrophages, which can activate the antitumor immunity and revive the tumor-killing capability in the HCC tumor microenvironment [32, 33]. We further compared the expression of different immune checkpoints reported in the previous study [33] between PTEN high- and low-expression groups. We found that in the PTEN low-expression group, there was a significant decrease in the expression of some immune checkpoints, including CD276, CD274, NRP1, and the TNFRSF family, that are closely involved in immunosuppressive signals in HCC tumor (Figure 2(d)). These results indicated that PTEN expression in the HCC tumor microenvironment might regulate the immunosuppressive signals and drive the antitumor immune cells' infiltration into HCC tumor tissues.

**Figure 3:**
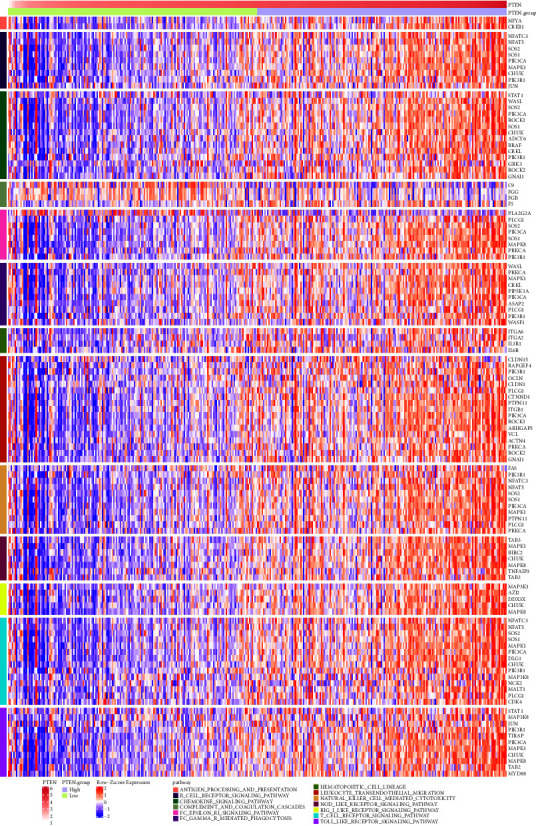
Comparing the enrichment of 13 immune-related pathways in high and low PTEN expression groups. The upper row axis indicates the PTEN expression level and PTEN expression groups. Immune-related pathways were differed by different colors, and the genes of each pathway were indicated on the right.

We therefore extracted the genes of immune-related pathways and found that with the increase of PTEN gene expression, the gene expression of most immune-related pathways also increased (Figure 3), suggesting a close correlation between PTEN gene expression and immune-related pathways. Further exploration of the relationship between PTEN expression and immunosuppressive or immune-activated signaling pathways was necessary to better predict clinical outcome.

### 3.3. The Relationship between PTEN Expression and Autophagy

**Figure 4:**
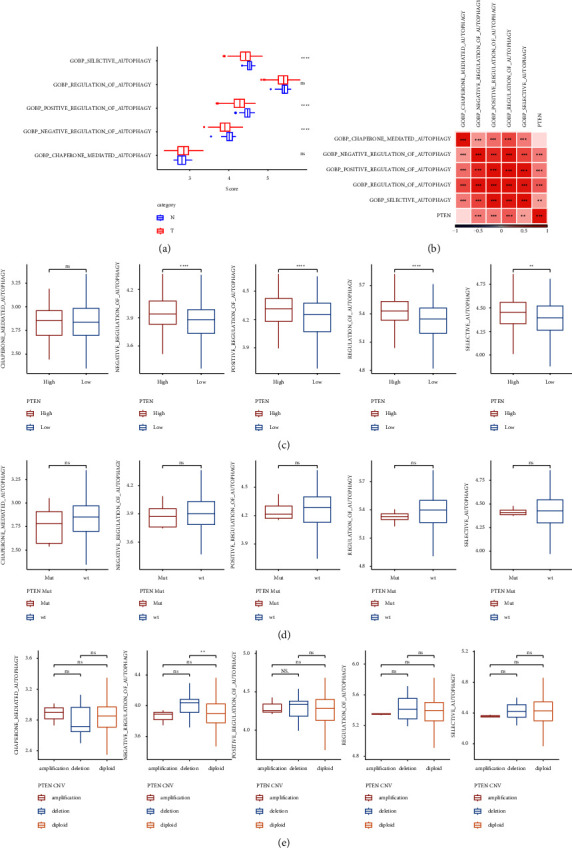
(a) Comparison of autophagy-related pathway scores in tumor tissue and paracancerous tissue; (b) correlation analysis of autophagy-related pathway scores and PTEN gene expression; (c) comparison of autophagy-related scores between high and low PTEN gene expression groups; (d) comparison of autophagy-related scores of the PTEN gene with or without SNV mutation samples; and (e) comparison of autophagy-related scores of the PTEN gene with CNV amplification, deletion, and diploid groups.

It has been reported that PTEN-L (PTEN*α*) is a novel phosphatase that mediates ubiquitin dephosphorylation and can inhibit PINK1/Parkin-mediated mitophagy [34]. Therefore, we further extracted five autophagy-related pathways and calculated the autophagy-related scores of these pathways. Compared to the paracancerous tissues, the scores of several signaling pathways, including the selective autophagy pathway and positive/negative regulation of the autophagy pathway, were significantly lower in the tumor tissues (Figure 4(a)). Moreover, we found that these autophagy-related pathways were significantly positively correlated with PTEN gene expression by performing correlation analysis (Figure 4(b)). To explore the relationship between the expression of the PTEN gene and autophagy, we divided the high- and low-expression groups according to the median expression of the PTEN gene and found that the high-expression group showed a higher autophagy score compared to the low-expression group, which was consistent with the results in Figure 4(b) (Figure 4(c)). The relationship between SNV mutation and CNV mutation of PTEN and autophagy was further analyzed. There was no significant difference between SNV mutation and CNV mutation of PTEN and the autophagy score (Figures 4(d) and 4(e)). These results indicated that PTEN may play a role in the autophagy-related signaling pathways and their function in HCC tumor progression.

### 3.4. Screening Gene Sets Related to Both PTEN and Autophagy

**Figure 5:**
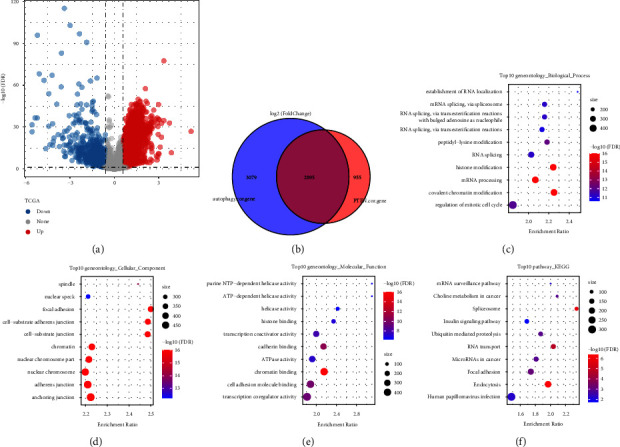
(a) The volcano plot of differential analysis of tumor tissue and paracancerous tissue in the TCGA dataset; (b) the venn plot of PTEN-related DEGs and autophagy-related genes; (c) the dot plot of BP enrichment analysis of shared genes; (d) the dot plot of the CC enrichment analysis of the shared gene; (e) the dot plot of the MF enrichment analysis of the shared gene; and (f) the dot plot of the KEGG enrichment analysis of the shared gene.

From the previous analysis, we found that the PTEN gene was associated with autophagy-related pathways, tumorigenesis, and tumor progression. Therefore, we analyzed the gene expression between tumor tissues and paracancerous tissues and identified differentially expressed genes (DEGs) between two groups (|log 2(fold change, FC)| > 1.5; false discovery rate (FDR) < 0.05) (Figure 5(a)). Within these DEGs between tumor and paracancerous tissues, there were 3818 and 32 genes positively and negatively correlated with PTEN expression, respectively. Moreover, a total of 5974 genes were found to be related to autophagy pathways. Through overlap analysis, we finally found a total of 2895 genes associated with both PTEN and autophagy (Figure 5(b)). Next, we performed GO and KEGG enrichment analyses [35] on the 2895 key genes package and found 23 pathways were enriched. Figures 5(c)–5(f) showed the visualization of the top 10 entries of the enrichment analysis. These enriched pathways and genes were closely related to the function of autophagy and PTEN-related signaling pathways that might affect tumor progression and clinical outcomes.

### 3.5. Construction of the PTEN-Autophagy Risk Model

**Figure 6:**
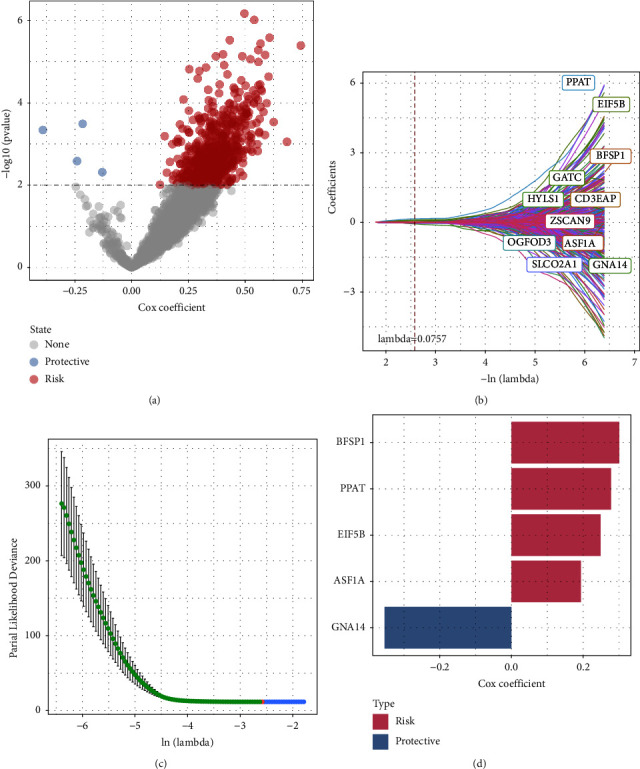
(a) A total of 2895 promising candidates were identified among the DEGs; (b) the trajectory of each independent variable with lambda; (c) confidence interval under lambda; and (d) multivariate Cox analysis and the coefficient of prognosis-related genes.

Next, we screened a total of 738 genes related to prognosis, of which 4 genes were protective and 734 genes were risk factors (Figure 6(a)). We further compressed these 738 genes using LASSO regression to reduce the number of genes contained in the risk model. Firstly, the change trajectory of each independent variable was analyzed (Figure 6(b)). With the gradual increase of lambda, the number of independent variable coefficients tending to 0 gradually increased. We used 10-fold cross-validation to build the model. As shown in Figure 6(c), the confidence interval under each lambda was analyzed and the model reached the optimal value when lambda was 0.0757. We selected 11 genes and further carried out stepwise multivariate regression analysis. As shown in Figure 6(d), we finally identified 5 genes, including BFSP1, PPAT, EIF5B, ASF1A, and GNA14, as prognostic genes related to PTEN and autophagy. The risk model was defined as follows: risk score = 0.194 ∗ ASF1A + 0.301 ∗ BFSP1 + 0.249 ∗ EIF5B − 0.354 ∗ GNA14 + 0.278 ∗ PPAT.

**Figure 7:**
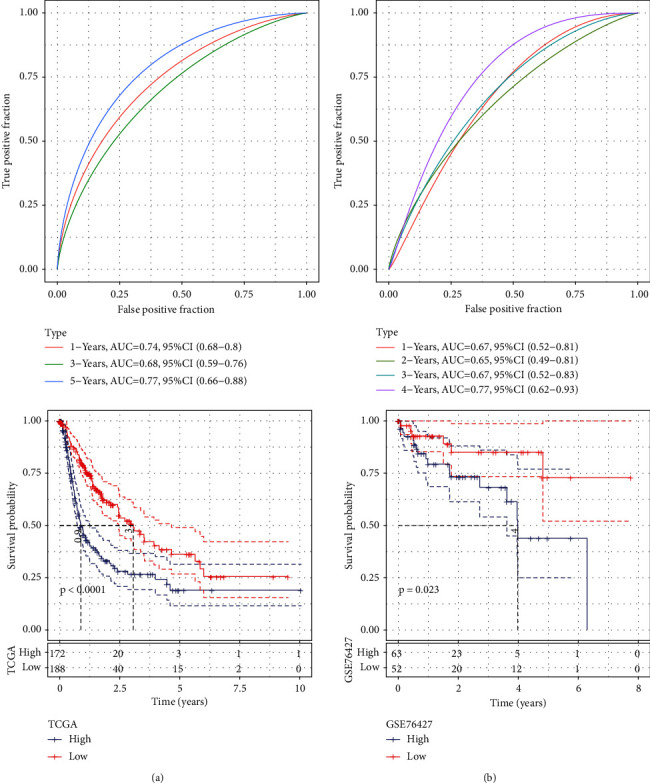
(a) ROC curve and the KM curve of the 5-gene model in the TCGA dataset (*n*-high = 172 and *n*-low = 188); (b) the ROC curve and the KM curve of the 5-gene model in the GSE76427 dataset (*n*-high = 63 and *n*-low = 52).

We then used the TCGA dataset as the training data set and calculated the risk score of each sample based on the expression levels of 5 genes. We analyzed the classification efficiency of the risk model in predicting 1, 3, and 5-year prognosis, respectively, and the time-dependent ROC curves (AUC) reached 0.7 in 1 and 5 years, indicating a strong predictive ability of this risk model. Then, the risk score was converted to a *z*-score, and the samples with a *z*-score greater than zero were divided into a high-risk group; otherwise, they were assigned to a low-risk group. As shown in Figure 7(a), the low-risk group showed a prolonged survival time compared to the high-risk group. We also used the same method to verify the GSE76427 independent dataset and observed similar survival benefits in the low-risk group (Figure 7(b)), indicating that the risk model had high predictivity.

### 3.6. Performance Comparison between PTEN-Autophagy.RS and TIDE

**Figure 8:**
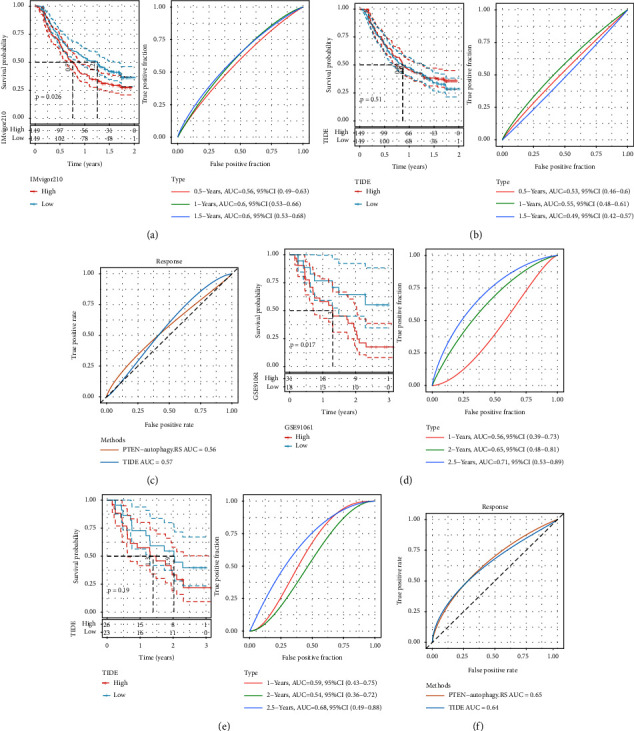
(a) KM curve and the ROC curve of PTEN-autophagy. RS in the IMvigor210 dataset (*n*-high = 149 and *n*-low = 149); (b) the KM curve and the ROC curve of immunotherapy response predicted by TIDE in the IMvigor210 dataset (*n*-high = 149 and *n*-low = 149); (c) the ROC curve of PTEN-autophagy. RS and TIDE on the immunotherapy effect in the IMvigor210 dataset; (d) the KM curve and the ROC curve of PTEN-autophagy. RS in the GSE91061 dataset (*n*-high = 31 and *n*-low = 18); (e) the KM curve and the ROC curve of immunotherapy response predicted by TIDE in the GSE91061 dataset (*n*-high = 26 and *n*-low = 23); and (f) ROC curves of immunotherapy effects of PTEN-autophagy. RS and TIDE in the GSE91061 dataset.

We then collected the clinical data after immunotherapy (IMvigor210 and GSE91061) and calculated the PTEN-autophagy.RS for the samples after immunotherapy. The online tool TIDE was used to evaluate the TIDE score of the immunotherapy effect. As shown in Figures 8(a) and 8(d), we found the poor prognosis in the high-risk group. We next compared the response to immunotherapy predicted by TIDE between the two datasets and found that there was no significant difference in the response (Figures 8(b) and 8(e)). We further calculated the PTEN-autophagy.RS and the AUC of TIDE on the effect of immunotherapy. The effect of PTEN-autophagy.RS on the immunotherapy was better than that of TIDE in the GSE91061 dataset (Figures 8(c) and 8(f)). It was basically close to the effect of TIDE in the IMvigor210 dataset. Overall, the PTEN-autophagy.RS model we constructed was better than TIDE in the immunotherapy effect.

### 3.7. PTEN-Autophagy.RS Combined with Clinicopathological Features to Further Improve the Prognostic Model and Survival Prediction

**Figure 9:**
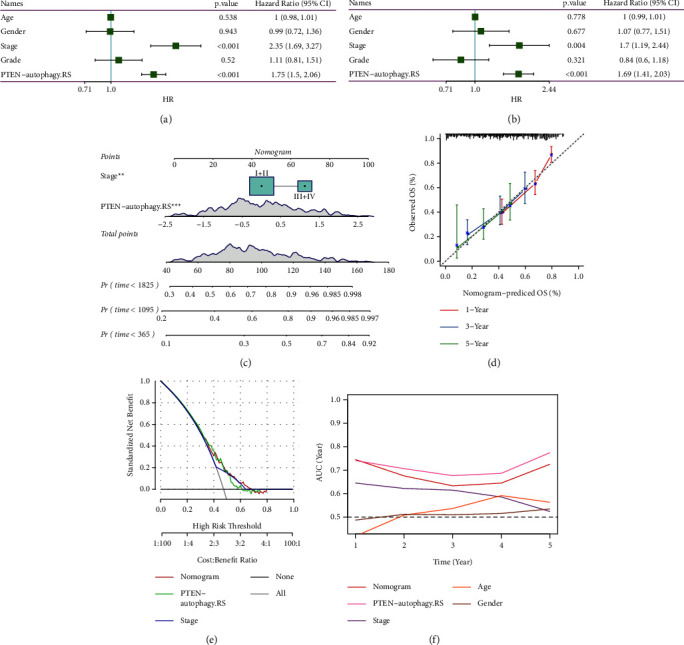
(a-b) Univariate and multivariate Cox analyses of PTEN-autophagy. RS and clinicopathological features; (c) nomogram model construction for predicting the survival probability of HCC patients; (d) calibration curves for 1, 3, and 5 years of the nomogram; (e) decision curve analysis for the nomogram; and (f) compared with other clinicopathological features, the nomogram exhibited the most powerful capacity for survival prediction.

Univariate and multivariate Cox regression analysis of the risk score and clinicopathological characteristics showed that PTEN-autophagy.RS was the most significant prognostic factor (Figures 9(a) and 9(b)). To quantify the risk assessment and survival probability of patients, we combined PTEN-autophagy.RS and other clinicopathological features. As shown in Figure 9(c), PTEN-autophagy.RS had the greatest impact on survival rate prediction. Furthermore, we used the calibration curve to evaluate the prediction accuracy of the model (Figure 9(d)). The predicted calibration curves of the three calibration points at 1, 3, and 5 years were nearly coincident with the standard curve, which suggested a strong predictive performance. In addition, we also used decision curve analysis (DCA) to evaluate the reliability of the model. As shown in Figures 9(e) and 9(f), the benefits of PTEN-autophagy.RS and nomogram were significantly higher than those of extreme curves. Compared with other clinicopathological features, PTEN-autophagy. RS showed the strongest survival prediction ability.

## 4. Discussion

HCC is the most common type of liver malignancies and shows increased incidence and mortality annually. The molecular mechanisms of HCC are complex, limiting the discovery of new targets and therapies in clinical research. Therefore, in this study, we explored the potential mechanisms related to HCC progression to predict prognosis. As demonstrated, PTEN is one of the tumor suppressor genes capable of negatively regulating the signaling pathways related to cancer progression. Although the function of the PTEN gene in immune regulation was found, the role of unexplored correlation between PTEN and immunity during HCC progression was still unknown. We therefore discovered their potential relationship and the significance of the PTEN gene in the tumor immune microenvironment. In addition to immune-relevant function, we also explored the correlation of the PTEN gene with autophagy and its related signaling pathways in HCC. Considering the importance of the PTEN gene and autophagy in HCC, we screened 5 genes including BFSP1, PPAT, EIF5B, ASF1A, and GNA14 associated with both the PTEN gene and autophagy. Among these genes, phosphoribosyl amidotransferase (PPAT) was demonstrated to serve as prognostic biomarkers for aggressive lung adenocarcinoma [36]. It was also reported that the expression of PPAT was upregulated during malignant progression [36]. PPAT could be used as one of the most promising therapeutic targets for its close correlation with patients' prognoses in many cancer types. Some studies have demonstrated that overexpression of EIF5B might induce PD-L1-related signaling pathways, which are frequent in lung adenocarcinomas and highly associated with a poor prognosis [37, 38]. The mechanisms of the PD-1/PD-L1 immune checkpoint, EIF5B, and PTEN genes in HCC could be further explored as therapeutic intervention. In addition, several studies found that ASF1A was overexpressed in human malignancies which was necessary for the proliferation of cancer cells [39–41]. ASF1A may also serve as a potential target in cancer therapy. The functional and clinical significance of ASF1A and its potential target against HCC should be considered.

We further established a PTEN-autophagy.RS prognostic model based on those genes and performed validation studies to assess the prediction efficiency in high- and low-risk groups. The results showed that the model can further improve the prognoses of patients. We used ESTIMATE and ssGSEA to estimate the tumor-infiltrating immune cells and immune scores for each HCC-patient sample and observed that the PTEN low-expression group had much higher scores in some immune cells, especially CD8 T cells, NK cells, B cells, and macrophages, which can activate the antitumor immunity and revive the tumor-killing capability in the HCC tumor microenvironment. The expression of different immune checkpoints was compared, and a significant decrease in the expression of some immune checkpoints was detected in HCC tumor. These results indicated that PTEN expression in the HCC tumor microenvironment might regulate the immunosuppressive signals and drive [1] antitumor immune cells infiltration into HCC tumor tissues. However, further exploration of the relationship between PTEN expression and immunosuppressive or immune-activated signaling pathways is necessary for a better understanding of the mechanism and predicting clinical outcome. Collectively, comparison on the data of immunotherapy with the performance of TIDE showed that the PTEN-autophagy.RS model we constructed was more sensitive than TIDE to the effects of immunotherapy.

The current findings should be further validated, especially for the association between PTEN and immune cell types in the tumor microenvironment, which can be a potential future research direction. Additionally, the comprehensive tumor microenvironment within HCC including the interaction between different cell types and autophagy is another promising direction in research.

## 5. Conclusion

In this study, we first evaluated the importance of the PTEN gene in HCC by analyzing the difference in PTEN gene expression in liver tumor tissue and adjacent tissue, CNV and SNV mutations, methylation, and other factors. Furthermore, the relationship between the PTEN gene and the tumor immune microenvironment in HCC was explored. Moreover, the correlation between autophagy and autophagy-related pathways for the importance of autophagy in tumorigenesis, PTEN gene expression, and mutation were also analyzed. Considering the role of the PTEN gene and autophagy in HCC, we screened 5 genes related to both the PTEN gene and autophagy using correlation analysis, univariate cox analysis, LASSO, and multivariate cox analysis. Finally, a PTEN-autophagy.RS model was constructed, and we demonstrated that the model can further improve the prognoses of patients. By comparing the data of immunotherapy with the performance of TIDE, it was further verified that the PTEN-autophagy.RS model we constructed was more sensitive than the TIDE score in response to immunotherapy.

## Data Availability

The data supporting the findings of the current study are available from the corresponding author upon request.The datasets generated and/or analyzed during the current study are available in the GSE76427 repository (https://www.ncbi.nlm.nih.gov/geo/query/acc.cgi?acc=GSE76427) and in the GSE91061 repository (https://www.ncbi.nlm.nih.gov/geo/query/acc.cgi?acc=GSE91061).
